# Retrospective Case Reports of Anemic Pregnant Women Receiving Intravenous Ferric Carboxymaltose: Experience from a Tertiary Hospital in Spain

**DOI:** 10.1155/2016/5060252

**Published:** 2016-10-20

**Authors:** Rafael Aporta Rodriguez, Mariola García Montero, Jose Pablo Lorente Aporta, Carolina Gallego Luque, Alfonso Chacón Mayor, Jose Aragón Ruiz, Virginia Torres Degayón, Claudia García Jimenez, Guadalupe Sanchez Sanchez

**Affiliations:** ^1^Hematology Department, Hospital Universitario de Ceuta, Ceuta, Spain; ^2^Gynecology and Obstetrics Department, Hospital Universitario de Ceuta, Ceuta, Spain; ^3^Pharmacy Department, Hospital Universitario de Ceuta, Ceuta, Spain; ^4^Dermatology Department, Hospital Universitario de Ceuta, Ceuta, Spain

## Abstract

Iron deficiency and iron deficiency anemia during pregnancy call for safe treatment options that raise maternal hemoglobin levels and counterbalance iron demand and blood volume expansion while minimizing risks for the growing fetus. This retrospective study describes experience with intravenous ferric carboxymaltose given to pregnant women in a tertiary hospital in Spain. In a 5-year period, 95 pregnant women who had pretreatment hemoglobin <10 g/dL and at least one time of ferric carboxymaltose administration during pregnancy were included. Main outcome measures were week of pregnancy at iron administration, Hb levels before and after treatment, neonatal 5-minute Apgar scores, and birth weight. The majority received one dose of ferric carboxymaltose (1000 mg iron) during advanced pregnancy (median 31 weeks; interquartile range [IQR]: 27; 37 weeks) with minor to no adverse outcomes. Overall, median Hb increased from 8.5 g/dL (8.1; 8.9 g/dL) before treatment to 11.0 g/dL (9.9; 11.7 g/dL) after treatment. Normal Apgar scores were observed in all 97 infants (median birth weights 3560 g, 3270, and 3798 g). Four women received ferric carboxymaltose in the first trimester and twenty-eight during the second trimester without adverse outcomes for mother or child. These cases add to the evidence that ferric carboxymaltose administration during pregnancy is effective and safe.

## 1. Introduction

Anemia is a global burden (prevalence 32.9% in 2010) [1], particularly during pregnancy, affecting up to 56% of pregnant women in nonindustrialized countries and around 20% in industrialized countries [2, 3]. Anemia during pregnancy is most frequently caused by iron deficiency [4], with other causes including uncorrected anemia due to heavy menstrual bleeding (i.e., low iron stores preconception) and maternal hemorrhage. In fact, the 2010 global burden of disease study calculated the years lost to disease related to anemia due to maternal hemorrhage as 1-2 years per 100,000 cases [5].

Iron needs during pregnancy are enhanced due to iron demands of the growing fetus and the expansion of maternal blood volume [6]. Iron deficiency with or without anemia during pregnancy has been associated with significant risks for maternal and fetal morbidity [7]. For the expecting mother, these include impaired physical function [8], increased cardiac failure, and related death [8–10]. Furthermore, iron deficiency anemia during pregnancy predisposes the mother to postpartum anemia, which is associated with postpartum depression [11], impaired physical function [12], and impaired lactation [13].

For the developing fetus, maternal iron deficiency anemia is associated with chronic placental insufficiency [14] and with an increased risk for preterm delivery and low-birth weight (LBW) [15]. In addition, maternal iron deficiency anemia predisposes infants to early developmental iron deficiency [16], which may have serious and long lasting consequences by causing changes in brain structure and function [17]. Relationships between maternal poor iron status, intrauterine growth restriction (IUGR), and preterm birth or LBW have been established [4, 18, 19].

The conservative approach to anemia management typically considers intravenous (i.v.) iron as “second line” to oral iron [19]. However, newer i.v. iron preparations such as ferric carboxymaltose have excellent efficacy and safety profiles and offer advantages over oral iron such as faster response and better tolerability [19]. The aim of this present study is to retrospectively investigate the use and outcomes from FCM administration in pregnant women in a tertiary hospital in Spain.

## 2. Materials and Methods

### 2.1. Study Design

Data for these retrospective case reports were obtained by trained hematology service staff from the electronic patient records of the Department of Obstetrics and Gynecology, Hospital Universitario de Ceuta, Spain. Pregnant women who were referred from the emergency or gynecology department to the hematology department between September 2010 and April 2015 and had initial pretreatment hemoglobin (Hb) <10 g/dL and at least one time of i.v. ferric carboxymaltose administration (Ferinject®, Vifor Pharma Ltd., Switzerland) during their pregnancy were included. The study was conducted in line with the Declaration of Helsinki and in compliance with all applicable local and national guidelines and regulations. All administration times of i.v. iron were preceded by a risk/benefit assessment.

### 2.2. Treatment Characteristics

Ferric carboxymaltose was given according to the approved posology, that is, a maximum weekly iron dose of 1000 mg (up to 20 mg/kg body weight) in a single infusion given over at least 15 minutes.

### 2.3. Outcome Measures

Depending on availability, anonymized data were collected on maternal age, week of pregnancy at the time of ferric carboxymaltose administration, dose, Hb levels before and after treatment, and any adverse maternal outcomes. Neonatal outcomes were the week of pregnancy at delivery (preterm defined as <37 weeks), 5-minute Apgar scores, and birth weight (in grams; LBW defined as <2500 g). No formal statistics were performed.

## 3. Results

In the five-year period, 95 pregnant women with anemia who were referred to the hematology department and received ferric carboxymaltose during pregnancy were identified. The majority (98%) were singleton pregnancies. The median maternal age was 30 years (interquartile range [IQR]: 24; 35). The median week of pregnancy at the time of first ferric carboxymaltose administration was 31st week (27; 37 weeks). Most women (90/95) received single ferric carboxymaltose administration; 83 received a single 1000 mg iron dose and seven a 500 mg iron dose. The median total dose was 1000 mg (IQR: 1000; 1000 mg; range: 500–2000 mg).

Pretreatment Hb levels were determined 0–5 days before ferric carboxymaltose treatment. Routine posttreatment laboratory control of Hb level was performed either between 7 and 14 days (*n* = 63 women treated in third trimester) or between 20 and 40 days (*n* = 32 women treated in first or second trimester). In the latter group, four women who were initially treated in the second trimester required at least one further ferric carboxymaltose treatment prior to delivery.

Overall, the median Hb increased from 8.5 g/dL (8.1; 8.9 g/dL) before treatment to 11.0 g/dL (9.9; 11.7 g/dL) after treatment (Table 1). Among women treated in the first and second trimester, median Hb levels increased by 3.6 g/dL and 3.1 g/dL within 20–40 days. Among women treated in the third trimester, median Hb levels increased by 1.6 g/dL within 7–14 days. Postpartum Hb levels were not available because women were referred to primary care physicians after delivery and not followed up by hospital obstetricians. Median serum ferritin increased from 5.5 *μ*g/L (4.0; 8.8 *μ*g/L) to 85.5 *μ*g/L (45.5; 107.5 *μ*g/L) and transferrin saturation from 12.1% (7.2; 25.0%) to 21.0% (17.3; 27.8%) (*n* = 20 women were treated during first and second trimester and with available pre- and posttreatment iron status parameters).

The records of all 95 women indicated that ferric carboxymaltose administration was well tolerated. No serious adverse reactions were recorded.

All 95 women were considered to have had a standard birthing experience within the expected norms of the institution. Four women had a planned Cesarean delivery.

The median week of pregnancy at delivery was 40th week (39; 41 weeks). All infants (*n* = 97) had a 5-minute Apgar score between 7 and 10, which indicates a healthy spontaneously breathing infant with minor to no complications at birth. Nearly all infants (98%) had a normal birth weight including two sets of twins; the median birth weight overall was 3560 g (3270; 3798 g).

### 3.1. First Trimester Cases

Four women received ferric carboxymaltose in their first trimester. All four women had very low Hb levels prior to treatment (<9 g/dL), indicating severe anemia, which was corrected (Hb > 11 g/dL) within 20 to 40 days after treatment. All four women had term deliveries (>37 weeks) and the infant Apgar scores and birth weights were within normal ranges (Apgar score 7 or higher; birth weight 2970–4030 g). No further information regarding any other outcomes or the overall situation of these women could be retrieved.

### 3.2. Preterm Delivery Cases

Three cases of preterm delivery were identified, all considered unrelated to ferric carboxymaltose administration by the obstetrician. All three infants had normal Apgar scores and birth weights. The first case required a 36-year-old woman to have an emergency Cesarean delivery at 34 weeks (birth weight, 2950 g). During the first month of pregnancy only, this patient was treated with acenocoumarol; treatment was changed to low molecular weight heparin due to deep vein thrombosis. A single ferric carboxymaltose dose (1000 mg iron) was administered at 19 weeks of pregnancy (Hb level, 0–5 days before treatment, 7.5 g/dL; Hb level, 20–40 days after treatment, 11.4 g/dL). In the second case, a 34-year-old mother had a spontaneous vaginal delivery at 36 weeks (birth weight, 2660 g); data on any other complications were not available. The first ferric carboxymaltose dose (500 mg iron) was administered at 26 weeks of pregnancy (Hb level, 0–5 days before treatment, 8.4 g/dL; Hb level, 20–40 days after treatment, 10.4 g/dL); second ferric carboxymaltose administration (500 mg iron) was given one week later. The last case was in a 30-year-old woman who was involved in an unrelated trauma at 36 weeks. The obstetrician confirmed that the trauma had no effect on the pregnancy (birth weight, 3000 g). A single ferric carboxymaltose dose (1000 mg iron) was administered at 26 weeks of pregnancy (Hb level, 0–5 days before treatment, 8.7 g/dL; Hb level, 20–40 days after treatment, 12.6 g/dL).

### 3.3. Low-Birth Weight Cases

Two cases of term LBW infants (<2500 g) were identified, delivered at 39 weeks (2380 g) and 37 weeks (2450 g) of pregnancy in women aged 40 and 38 years, respectively. Apgar scores were 8 or above for both infants. Each mother received a single ferric carboxymaltose treatment (1000 mg iron) late in the second trimester, both at 27 weeks of pregnancy. The pretreatment Hb levels were 8.1 and 8.7 g/dL, and the posttreatment Hb levels between 20 and 40 days after treatment were 11.0 g/dL and 11.5 g/dL, respectively. The LBW was considered unrelated to the iron administration. No further information regarding any other outcomes or the overall situation of these women could be retrieved.

## 4. Discussion

This retrospective case report describes experience with routine use of intravenous ferric carboxymaltose during pregnancy in a tertiary hospital in Spain, illustrating its effectiveness in raising maternal Hb levels. It is expected that additional increases in Hb levels occurred after the routine laboratory control for the majority of women. Such long-term increases may be smaller in magnitude but remain clinically meaningful (e.g., in terms of improved quality of life or fatigue control due to repletion of iron stores) [20, 21]. The treatment was generally safe and well tolerated; yet, mild and transient adverse events may not have been captured in the analyzed electronic patient records.

In the majority of cases, the moderate-to-severe anemia during pregnancy was quickly managed with minimal inconvenience. Prior experience in the hospital showed that oral iron is not sufficiently effective in such women. This is in line with other reports showing slow response to oral iron, likely due to low intestinal absorption and/or low adherence caused by high rates of side effects [4, 19]. Accordingly, oral iron is rather feasible to prevent potential (mild) iron deficiencies without anemia during pregnancy.

After treating seven women with 500 mg intravenous iron, the standard protocol for treatment of pregnant women with Hb levels <10 g/dL was changed to a single ferric carboxymaltose dose of 1000 mg iron. Ferric carboxymaltose was even administered the day before delivery in one woman without causing deleterious effects on the mother, during delivery, or the infant. Furthermore, for the four cases where ferric carboxymaltose administration was given in the first trimester, the Hb responses to iron treatment were considered excellent, affording the same safety margin typically reserved for second or third trimester administration, and their four infants were all healthy at birth.

Apart from the two LBW infants with almost normal birth weights (50 and 120 g below the cut-off), normal birth weights were observed and within the expected norms for the institution. These LBW infants were reported healthy at delivery as evidenced by normal Apgar scores and LBW was determined to be unrelated to iron administration. Other considerations as to the cause of LBW (e.g., associated factors such as maternal weight and parity) cannot be ruled out. These two mothers may have experienced an already prolonged period of anemia because the treatment was given late in the second trimester and the time to benefit from increased Hb levels (+2.9 and +2.8 g/dL) may have been too short. Additional studies with longer term follow-up of the developmental progress of infants born to mothers with anemia during pregnancy and the role of intravenous iron in this hospital are warranted.

Assessment of clinical endpoints other than laboratory values in pregnant women in prospective controlled trials evaluating treatments of anemia is scarce. Nevertheless, the severity of anemia during pregnancy is widely recognized as an important risk factor to maternal and fetal morbidity and mortality [4, 18]. Consensus recommendations define anemia during pregnancy as Hb level <10.5 g/dL (severe anemia < 9 g/dL), that is, the cut-off value to initiate therapy, with the choice of treatment depending not only on the trimester but also on the cause and severity of anemia [19, 22]. Furthermore, the recommendations support a pragmatic approach during pregnancy, particularly in cases where very low Hb values, unresponsiveness or undesirable side effects to oral iron, or refusal of transfusion may necessitate i.v. iron. Indeed, ferric carboxymaltose may be the preferred alternative [19].

A few studies have reported safe and effective use of ferric carboxymaltose for anemia correction in the second or third trimester or in the postpartum period [23–25]. When compared with i.v. iron sucrose, ferric carboxymaltose had a similar safety profile while having the practical advantage of allowing a higher iron dose in one time of administration (minimizing repeated administration times and increasing patient comfort) [24]. Notably, Froessler et al. demonstrated in a prospective observational study of 65 anemic pregnant women in Australia who received ferric carboxymaltose that Hb levels significantly increased above baseline levels and 66% of women reported an improvement of their wellbeing with mostly minor and self-limiting side effects. The cases described in this report are also in line with the retrospective case-control study from the Netherlands reporting similar significant increases in maternal Hb levels above baseline and low rates of adverse outcomes [23].

The most frequently used parameter to assess maternal iron status (and thus iron demands) is serum ferritin concentration. Although there may be limitations to the use of ferritin as marker of iron status during transient inflammatory reactions (raising ferritin to falsely normal or even elevated levels), in general, a more rigorous approach to the diagnosis and treatment of iron deficiency anemia during pregnancy with additional laboratory markers such as ferritin is advisable. While iron deficiency is the usual suspect for IUGR in developed countries, iron status is rarely assessed during pregnancy and iron supplementation is often based solely on Hb levels, as in the cases reported here. Due to the retrospective nature of this study, only few data were available for all the 95 cases that could more precisely describe maternal baseline comorbidities, iron status, and overall condition during pregnancy (e.g., whole blood counts, serum ferritin, and IUGR).

In summary, within the limitations of these retrospective case reports, this hospital's experience adds to the evidence that timely management of moderate-to-severe anemia during pregnancy with i.v. iron is feasible and, with the necessary precautions, potentially safe in the first trimester. These results are consistent with the available reports of the safe and effective use of ferric carboxymaltose in the treatment of iron deficiency anemia in pregnancy.

## Figures and Tables

**Table 1:** Response to intravenous ferric carboxymaltose.

	*n* (%)	Maternal age, years	Pregnancy week at time of first FCM treatment	Pre-treatment Hb, g/dL	Post-treatment Hb, g/dL^#^
First trimester	4 (4.2%)	38 (34; 41)	11 (9; 14)	8.0 (7.7; 8.3)	11.6 (11.4; 11.7)
Second trimester^*∗*^	28 (29.5%)	31 (28; 36)	26 (24; 27)	8.6 (8.0; 8.8)	11.7 (11.3; 12.5)
Third trimester^†^	63 (66.3%)	28 (23; 34)	35 (31; 38)	8.6 (8.1; 8.9)	10.2 (9.6; 11.0)

*All*	*95 (100%)*	*30 (24; 35)*	*31 (27; 37)*	*8.5 (8.1; 8.9)*	*11.0 (9.9; 11.7)*

Data shown as median (IQR) or *n* (%).

^*∗*^Three women received at least one further treatment (two women received two FCM administrations and one woman received three FCM administrations).

^†^One woman received two FCM administrations of 1000 mg iron three weeks apart.

^#^Routine laboratory control was performed either between 7 and 14 days (women in third trimester of pregnancy) or between 20 and 40 days (women in first or second trimester of pregnancy) after treatment.

FCM, ferric carboxymaltose; Hb, hemoglobin; IQR, interquartile range.

## References

[1] Kassebaum N. J., Bertozzi-Villa A., Coggeshall M. S. (2013). Global, regional, and national levels and causes of maternal mortality during 1990–2013: a systematic analysis for the Global Burden of Disease Study 2013. *The Lancet*.

[2] World Health Organization (2008). *Worldwide Prevalence of Anaemia 1993–2005*.

[3] Bencaiova G., Burkhardt T., Breymann C. (2012). Anemia—prevalence and risk factors in pregnancy. *European Journal of Internal Medicine*.

[4] Milman N. (2008). Prepartum anaemia: prevention and treatment. *Annals of Hematology*.

[5] Vos T., Flaxman A. D., Naghavi M. (2012). Years lived with disability (YLDs) for 1160 sequelae of 289 diseases and injuries 1990–2010: a systematic analysis for the Global Burden of Disease study 2010. *The Lancet*.

[6] Milman N. (2006). Iron and pregnancy—a delicate balance. *Annals of Hematology*.

[7] Bergmann R. L., Dudenhausen J. W., Ennen J. C. (2009). Diagnosis and treatment of iron deficiency and anaemia during pregnancy and post partum. *Geburtshilfe und Frauenheilkunde*.

[8] Viteri F. E. (1994). The consequences of iron deficiency and anaemia in pregnancy on maternal health, the foetus and the infant. *SCN News*.

[9] Villar J., Merialdi M., Gülmezoglu A. M. (2003). Nutritional interventions during pregnancy for the prevention or treatment of maternal morbidity and preterm delivery: an overview of randomized controlled trials. *The Journal of Nutrition*.

[10] Reveiz L., Gyte G. M., Cuervo L. G., Casasbuenas A. (2011). Treatments for iron-deficiency anaemia in pregnancy. *Cochrane Database of Systematic Reviews*.

[11] Corwin E. J., Murray-Kolb L. E., Beard J. L. (2003). Low hemoglobin level is a risk factor for postpartum depression. *Journal of Nutrition*.

[12] Van Wyck D. B., Martens M. G., Seid M. H., Baker J. B., Mangione A. (2007). Intravenous ferric carboxymaltose compared with oral iron in the treatment of postpartum anemia: a randomized controlled trial. *Obstetrics and Gynecology*.

[13] Breymann C., Hugh R. (2008). *Anaemia in Pregnancy and the Puerperium*.

[14] Pavlova T. V., Petrukhin V. A., Zhilyaeva O. D., Nadezhdin S. V. (2007). Placental morphology in pregnancy in the presence of iron-deficiency anemia. *Arkhiv Patologii*.

[15] Scholl T. O. (2005). Iron status during pregnancy: setting the stage for mother and infant. *The American Journal of Clinical Nutrition*.

[16] Allen L. H. (2000). Anemia and iron deficiency: effects on pregnancy outcome. *The American Journal of Clinical Nutrition*.

[17] Beard J. L. (2008). Why iron deficiency is important in infant development. *The Journal of Nutrition*.

[18] Breymann C. (2002). Iron supplementation during pregnancy. *Fetal and Maternal Medicine Review*.

[19] Breymann C., Honegger C., Holzgreve W., Surbek D. (2010). Diagnosis and treatment of iron-deficiency anaemia during pregnancy and postpartum. *Archives of Gynecology and Obstetrics*.

[20] Favrat B., Balck K., Breymann C. (2014). Evaluation of a single dose of ferric carboxymaltose in fatigued, iron-deficient women—PREFER a randomized, placebo-controlled study. *PLoS ONE*.

[21] Brownlie T., Utermohlen V., Hinton P. S., Haas J. D. (2004). Tissue iron deficiency without anemia impairs adaptation in endurance capacity after aerobic training in previously untrained women. *American Journal of Clinical Nutrition*.

[22] Breymann C., Bian X.-M., Blanco-Capito L. R., Chong C., Mahmud G., Rehman R. (2011). Expert recommendations for the diagnosis and treatment of iron-deficiency anemia during pregnancy and the postpartum period in the Asia-Pacific region. *Journal of Perinatal Medicine*.

[23] Pels A., Ganzevoort W. (2015). Safety and efficacy of ferric carboxymaltose in anemic pregnant women: A Retrospective Case Control Study. *Obstetrics and Gynecology International*.

[24] Christoph P., Schuller C., Studer H., Irion O., De Tejada B. M., Surbek D. (2012). Intravenous iron treatment in pregnancy: comparison of high-dose ferric carboxymaltose vs. iron sucrose. *Journal of Perinatal Medicine*.

[25] Froessler B., Collingwood J., Hodyl N. A., Dekker G. (2014). Intravenous ferric carboxymaltose for anaemia in pregnancy. *BMC Pregnancy and Childbirth*.

